# Hadamard Matrix Slicing Single‐Pixel Imaging: Establishing a Linear‐Array‐Inspired Paradigm for *N*‐fold Acceleration Single‐Pixel Imaging

**DOI:** 10.1002/advs.75643

**Published:** 2026-05-12

**Authors:** Xiaoxue Li, Jieting Hou, Qizheng Zhao, Yiqi Jia, Xiang Zhong, Mengchao Ma, Huaxia Deng, Xinglong Gong, Ziwei Wang

**Affiliations:** ^1^ Anhui Province Key Laboratory of Measuring Theory and Precision Instrument School of Instrument Science and Opto‐Electronics Engineering Hefei University of Technology Hefei Anhui P. R. China; ^2^ CAS Key Laboratory of Mechanical Behavior and Design of Materials Department of Modern Mechanics University of Science and Technology of China Hefei Anhui P. R. China; ^3^ School of Engineering Lancaster University Lancaster UK

**Keywords:** hadamard matrix slicing, linear array scanning, single‐pixel imaging

## Abstract

Linear array detectors dominate fields like industrial inspection owing to their efficient push‐broom scanning modes. However, Single‐Pixel Imaging (SPI) remains constrained by global 2D modulation, where the requisite pattern count scales quadratically with resolution. This imposes severe bottlenecks on data throughput and modulation duration. To overcome these limitations, Hadamard Matrix Slicing Single‐Pixel Imaging (HMS‐SPI) is proposed, which establishes an efficient 1D imaging paradigm analogous to linear array sensors. By mathematically slicing the traditional 2D Hadamard matrix into 1D encoding vectors, HMS‐SPI reduces the measurement patterns required for an N×N image by a factor of N while maintaining full sampling. Experimentally, a 768×512 image is reconstructed from 1,024 measurements in 12.8 seconds at 80 Hz. Furthermore, theoretical analysis demonstrates a potential 51.2 milliseconds acquisition time at a 20 kHz modulation limit. This approach drastically reduces memory consumption and pattern loading latency. By emulating the efficiency of push‐broom scanning, HMS‐SPI provides a transformative solution for high‐throughput SPI applications.

## Graphical abstract

To overcome two‐dimensional modulation bottlenecks, Hadamard Matrix Slicing Single‐Pixel Imaging (HMS‐SPI) establishes an efficient one‐dimensional imaging paradigm. By slicing the traditional Hadamard matrix into one‐dimensional encoding vectors and spatially expanding them, the required measurement patterns decrease by a factor of N. HMS‐SPI drastically shortens acquisition time and reduces memory consumption, providing a transformative solution for high‐throughput applications.

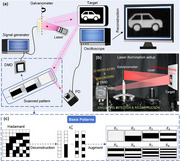

## 1 Introduction

Functioning in a push‐broom scanning configuration, line‐scan detectors [1, 2] are distinguished by their ability to generate high‐definition 2D imagery while maintaining superior temporal efficiency and high data throughput. Consequently, this architecture has been extensively adopted in modern machine vision and Earth observation systems. Conversely, SPI represents a computational imaging paradigm characterized by a broad spectral range, robust noise immunity, and superior adaptability to low‐light conditions [3, 4]. By enabling non‐local image reconstruction via a single‐point detector [5, 6, 7, 8, 9, 10], SPI is particularly well‐suited for scenarios that remain challenging for conventional imaging systems. Nevertheless, contemporary SPI techniques predominantly adhere to an area‐array paradigm, employing a Digital Micromirror Device (DMD) to perform global structured illumination modulation across the entire 2D target scene [11, 12, 13, 14, 15]. As a result, the data acquisition efficiency of such systems has historically been constrained by the limitations inherent in 2D modulation schemes.

While 2D encoding strategies enable target recovery via non‐local image reconstruction algorithms, they introduce a significant bottleneck. Specifically, to reconstruct an image with a resolution of N×N, traditional SPI based on Hadamard or Fourier bases typically requires measurements on the order of $N^{2}$. As the resolution increases, the number of required patterns scales quadratically, which not only imposes stringent requirements on the refresh rate of the DMD but also results in massive storage overhead for patterns and prolonged pre‐loading latencies [16]. To address the above challenges, various techniques have been extensively explored, including photon time stretching (PTS) techniques [17], spectral pulse shaping techniques [18, 19, 20, 21, 22, 23], quadrant photodiode detectors [24, 25], compressed sensing [26, 27, 28], 1D imaging [29], ghost imaging [30, 31, 32, 33], and deep learning methods [34, 35, 36, 37, 38]. Moreover, there is an increasing amount of recent research utilizing multi‐dimensional optical manipulation for high‐efficiency image reconstruction. In complex 3D measurement scenarios, Tan et al. [39] and Su et al. [40] propose a region projection technique based on spectral division multiplexing (SDM) to effectively suppress multi‐path interference caused by mutual reflections, achieving high‐fidelity and high‐efficiency optical reconstruction. However, the field of SPI remains devoid of a line‐scan mechanism comparable to a linear array CCD, a mechanism that significantly mitigates data redundancy by reducing the dimensionality.

To address the aforementioned challenges and bridge the gap in efficient 1D scanning SPI, this paper proposes an innovative HMS‐SPI method. This approach circumvents the reliance of traditional SPI on 2D matrix patterns. Through the co‐design of optical hardware and coding algorithms, it achieves an efficient 1D‐to‐2D conversion, thereby drastically reducing the DMD modulation time. Specifically, a linear laser source is utilized in conjunction with a high‐precision galvanometer to establish a periodic scanning optical path, enabling row‐by‐row physical scanning of the target. Simultaneously, the traditional 2D Hadamard matrix undergoes a slicing operation to be reconfigured into 1D modulation patterns adapted for line scanning. This design enables the system to process exclusively 1D information during each DMD modulation frame, thereby reducing the order of measurement bases from $N^{2}$ to 2N. Experimental results demonstrate that the linear scanning mechanism requires only 1,024 measurements to reconstruct a 768×512 image in 12.8 seconds at an 80 Hz modulation rate. Theoretically, matching the scanning galvanometer to the higher frame rates of the DMD can drastically reduce the modulation time, enabling the reconstruction of a 768×512 image in just 51.2 milliseconds at 20 kHz. This method significantly lowers DMD memory consumption and breaks the square‐image restriction, marking a qualitative leap in SPI efficiency.

## 2 Results

### 2.1 Principles

**FIGURE 1:**
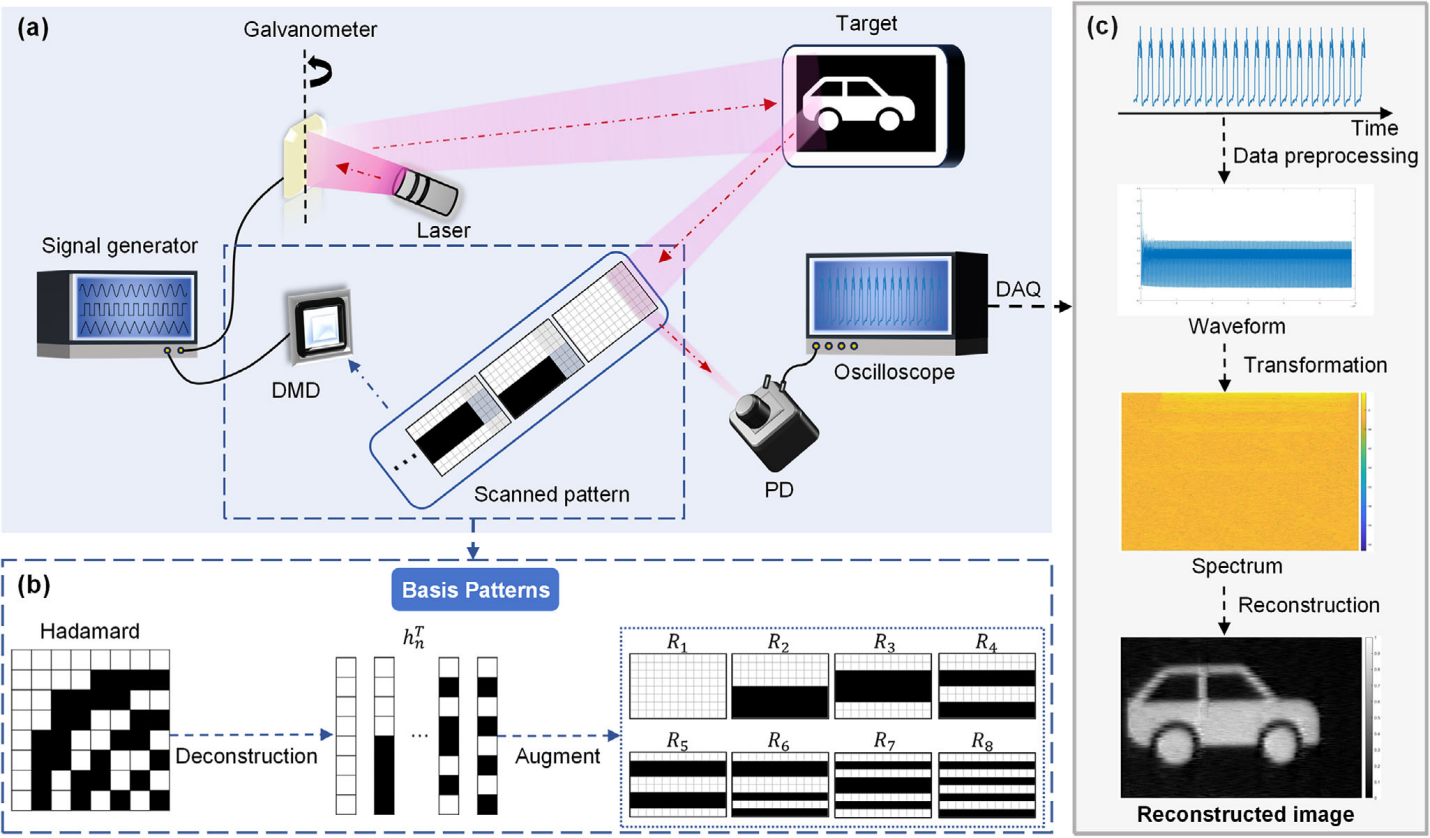
Principle of the HMS‐SPI system. (a) Schematic diagram of the proposed system. PD, Photodetector; DAQ, Data Acquisition. (b) Principle of the 8th‐order Hadamard matrix slicing reconstruction. (c) Flowchart of the image reconstruction process.

The schematic diagram of the proposed HMS‐SPI method is shown in Figure 1a. The imaging procedure is divided into a modulation process and a demodulation process. Employing an SPI configuration, the modulation process involves periodically illuminating the target object using a line‐shaped laser source in conjunction with a precision optical scanning path. The pre‐designed, extended 1D coded patterns are transmitted to the DMD controller, which acts as the modulator. Subsequently, the light reflected from the object propagates to the surface of the spatial light modulator, where the image is modulated. In the demodulation process, the corresponding series of modulated light intensity values are captured by a single‐pixel photodetector. These values are converted into electrical signals, which are then recorded and stored by an oscilloscope. Finally, these signals are processed and demodulated by a computer to reconstruct the target image. The principle of the demodulation process is depicted in Figure 1c.

The coded illumination pattern used in the Hadamard single‐pixel imaging technique is the Hadamard matrix. Consequently, the resolution of the reconstructed image is typically n×n pixels, where n is a power of two. In this work, we slice and analyze the Hadamard matrix and propose a new modulation method for imaging. This method makes it possible to reconstruct rectangular target areas in a single process. Furthermore, it significantly reduces the number of patterns required for image reconstruction. This reduction alleviates the memory and loading demands of the preloading process, allowing for higher imaging rates.

Any two rows (or columns) of the Hadamard matrix are orthogonal, which ensures that the signals of different bases are still mutually independent in their transformed representations. $H_{n}$ represents the Hadamard matrix, denoted as follows:

(1) $$H_{n}={\left[h_{1}^{T},h_{2}^{T},\cdots ,h_{n}^{T}\right]}^{T},$$

where $h_{n}$ is the n‐th row vector of the Hadamard matrix. We design basis patterns that conform to the scanning based on the Walsh sequence feature of the Hadamard matrix. First, as illustrated by the ”Deconstruction” step in Figure 1b, the original n‐order Hadamard patterns are sliced in rows (or columns) to obtain n vectors $H_{i}^{*}$ (i=1,2,⋯,n):

(2) $$H_{1}^{*}=\left[h_{1}^{T}\right],H_{2}^{*}=\left[h_{2}^{T}\right],\cdots ,H_{n}^{*}=\left[h_{n}^{T}\right],$$

Next, corresponding to the ”Augment” step, the 1D vectors are spatially expanded to match the target. Reconstruction of the m slices yields the matrix $R_{n}$:

(3) $$\begin{array}{cc} R_{1} & =[\underset{m}{\underset{︸}{H_{1}^{*},H_{1}^{*},\cdots ,H_{1}^{*}}}],\\ R_{2} & =[\underset{m}{\underset{︸}{H_{2}^{*},H_{2}^{*},\cdots ,H_{2}^{*}}}],\\ & \vdots \\ R_{n} & =[\underset{m}{\underset{︸}{H_{n}^{*},H_{n}^{*},\cdots ,H_{n}^{*}}}], \end{array}$$

The number of slices in the reconstruction matrix, denoted as m, is determined by the lateral resolution of the target image. Each scanning basis pattern composed of the reconstructed Hadamard slices consists of m column vectors, denoted as $R_{i}$:

(4) $$R_{i}=\left[r_{i},r_{i},\cdots ,r_{i}\right]=\left[H_{i}^{*},H_{i}^{*},\cdots ,H_{i}^{*}\right];\;i=1,2,\cdots ,n,$$

Hadamard‐based Single‐Pixel Imaging (HSI) relies on the orthogonal Hadamard transform [38]. It reconstructs the target image by acquiring the Hadamard spectrum and subsequently applying the inverse Hadamard transform. Figure 1b shows an example of the scanning basis patterns designed using an 8th‐order Hadamard matrix and the modulation principle. The resolution of the scanned basis pattern is the same as the target, and the modulation process is expressed as follows:

(5) $$q_{ai}=P_{a}^{T}\cdot r_{i};\;a=1,2,\cdots ,m;\;i=1,2,\cdots ,n,$$

The measured light intensity $q_{ai}$ is obtained by modulating the a‐th column of the target, $P_{a}$, with the corresponding code $r_{i}$ from each scanned basis pattern. The target resolution is m×n, meaning that $P_{a}$ is an n×1 column vector.

Differential measurements are often used to enhance the immunity of Hadamard single‐pixel imaging. They allow the individual Hadamard coefficients Q(u,v) to be obtained in a differential measurement.

(6) $$Q\left(u,v\right)=Y_{+}-Y_{-},$$

where $Y_{+}$ and $Y_{-}$ are the light intensity values corresponding to the target after it has been modulated by a positive $S_{+}$ and a negative $S_{-}$ Hadamard scanning basis pattern, respectively, which are denoted as:

(7) $$S_{+}=\left(1+h_{n}\right)/2,$$

(8) $$S_{-}=\left(1-h_{n}\right)/2,$$

(9) $$Y\left(n\right)=\sum_{(n-1)\cdot i\cdot k+l}^{n\cdot i\cdot k}y\left(x\right),$$

where Y(n) is a set of light intensity values, i·k is the number of measurements corresponding to each pattern, i is the value of the lateral resolution m of the pattern, k is the number of repetitive acquisitions of the light intensity values during each modulation, n is the number of modulations corresponding to the n‐th coded pattern and the target information, l is the number of rounded off light intensity values belonging to the characteristics of the DMD, and y(x) denotes the individual discrete light intensity value recorded by the photodetector under the illumination of each encoded pattern.

After obtaining the Hadamard coefficients of the target image, the Hadamard spectrum matrix Q is obtained:

(10) $$Q_{a}=\left[q_{a1},q_{a2},q_{a3},\cdots ,q_{an}\right];\;a=1,2,\cdots ,m,$$

(11) $$Q={\left[Q_{1}^{T},Q_{2}^{T},Q_{3}^{T},\cdots ,Q_{m}^{T}\right]}^{T},$$

The target image P can be reconstructed by inverting the Hadamard spectrum. The principle of this reconstruction process is expressed as follows:

(12) $$P=Q\cdot H_{n},$$

Since the target area with m×n resolution can be decomposed into m vector groups, each of which is of size n×1, only 2n first‐order Hadamard pattern modulations are required for each 1/m target sequence. The process of vibratory dynamic scanning exactly traverses the m columns of the image, which is precisely why care needs to be taken in compressed sampling that the scanning basis pattern can only be varied in the transverse dimension but not reduced in the longitudinal dimension.

The proposed method provides a significant advantage in Hadamard single‐pixel imaging. To reconstruct an image of m×n resolution, this method utilizes only 2n patterns, which reduces the demand for pre‐loaded patterns by a factor of m relative to traditional techniques and leads to a substantial decrease in imaging time. Moreover, the row‐by‐row modulation scheme frees the imaging resolution from the constraint of square target areas and removes the necessity for the lateral resolution to be an integer power of two.

### 2.2 Simulations

**FIGURE 2:**
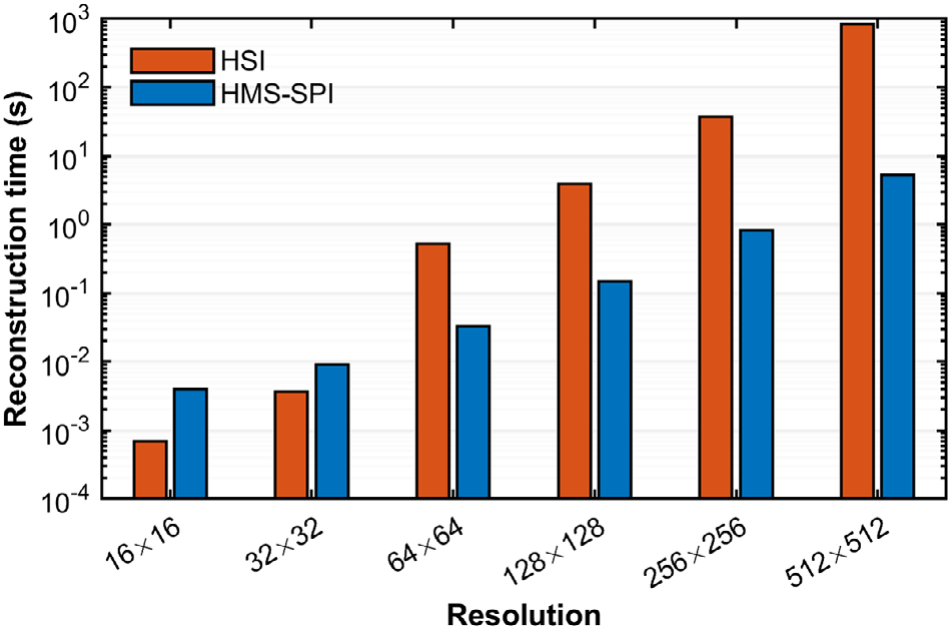
Comparison of computation times between the conventional HSI and the HMS‐SPI at different resolutions.

Conventional single‐pixel imaging methods employ an undersampling strategy to reduce the number of measurements, but this often leads to degraded imaging quality. Moreover, as image resolution increases, both the imaging time and computational demand escalate rapidly. Our proposed HMS‐SPI method offers significant advantages in the required number of patterns and overall computation time. For an image with a resolution of N×N, conventional HSI requires $2N^{2}$ patterns. In contrast, our method necessitates only 2N patterns, reducing the required number to 1/N of the original. We utilize MATLAB numerical simulations to compare the differences in computation time between the two methods. Figure 2 displays a bar chart of the computation time, where the vertical axis represents the logarithm of the reconstruction time, facilitating an intuitive comparison of the results.

The advantages of the proposed method are demonstrated through simulation experiments. Notably, starting from a 64×64 resolution, the computational time for conventional HSI increases sharply. As shown in Figure 2, our method holds a significant advantage in reconstruction speed. Specific reconstruction times for different image resolutions are presented in Note S1.

**FIGURE 3:**
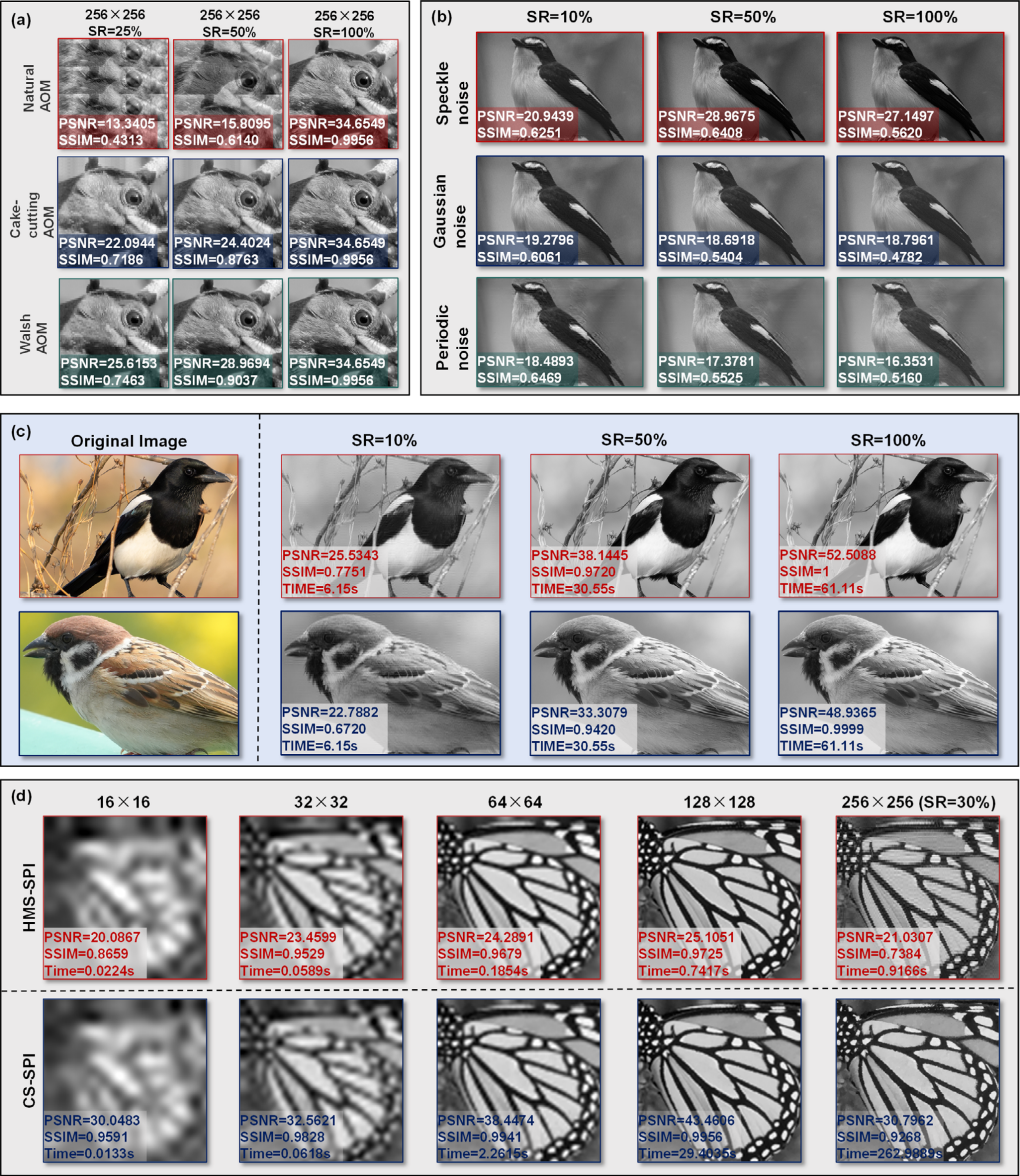
Summary of simulation results. (a) Simulation results for HMS‐SPI with different ordering schemes. (b) Simulation results for HMS‐SPI under different noise conditions. (c) Noise‐free simulation results for HMS‐SPI on two different target images. (d) Comparison of simulation reconstruction times between HMS‐SPI and Compressive Sensing (CS) SPI at different resolutions. The butterfly image used in (d) is sourced from the publicly available Set5 dataset [41].

In our simulation experiments, Hadamard matrices ordered by natural, cake‐cutting [42], and Walsh sequences are employed for the slicing reconstruction of the “barbet” image. Novel Hadamard measurement matrices are generated through the replication and extension of 1D vectors, and the image reconstruction performance of these three ordering schemes is validated. The reconstruction outcomes are evaluated using the Peak Signal‐to‐Noise Ratio (PSNR) and the Structural Similarity Index Measure (SSIM), two universally recognized metrics in the digital imaging domain, to identify the optimal matrix ordering scheme for HMS‐SPI. The quality assessment metrics are detailed in Note S5. The simulation results for HMS‐SPI, including the reconstructed images and corresponding evaluation metrics for each ordering scheme, are presented in Figure 3a. These results demonstrate that the HMS‐SPI framework based on the Walsh ordering yields the highest reconstruction quality. Consequently, this ordering scheme is adopted for all subsequent experiments in Hadamard matrix slicing single‐pixel imaging.

Figure 3c illustrates the image reconstruction results for a “magpie” and a “sparrow” at a resolution of 1565×1024 pixels under various sampling rates. The computation time for each reconstruction is under 62 s, demonstrating a significant advantage in terms of computational complexity during the simulation. As the sampling rate decreases, the image signal‐to‐noise ratio progressively degrades. At lower sampling rates, stripe‐like mosaic artifacts become apparent. Nevertheless, HMS‐SPI effectively preserves image details even at low sampling rates and remains unaffected by binarization errors.

HMS‐SPI employs a passive architecture, wherein a line‐shaped laser source and a high‐speed galvanometer periodically illuminate the target, and the light intensity signal is modulated by 1D encoding. This method is susceptible to three primary types of noise: speckle noise introduced by laser coherence, Gaussian noise from electronic components and environmental fluctuations, and periodic positional offsets caused by the mechanical characteristics of the scanning galvanometer. The reconstruction results from simulation experiments on the 1565×1024 pixel “flycatcher” image under different noise conditions are presented in Figure 3b. The simulation results indicate that Gaussian and periodic noise have a significant impact, causing a granular appearance or image artifacts. To enhance imaging quality, beyond post‐processing, it is crucial to control the experimental environment and ensure hardware synchronization. This helps mitigate the effects introduced by the optical path and hardware, while striking an optimal balance among resolution, speed, and quality.

For a quantitative comparison, image reconstruction simulations are conducted using both the proposed method and the TVAL3 compressive sensing algorithm. In these simulations, “butterfly” images of varying resolutions are reconstructed, with the simulation reconstruction time being recorded for each. The image reconstruction quality is quantitatively assessed using the commonly employed PSNR and SSIM metrics from the image processing field. The simulation results for both methods are displayed in Figure 3(d). The experimental results show that although the TVAL3 algorithm enhances image reconstruction quality through total variation regularization, achieving favorable PSNR and SSIM parameters, it struggles with the computational demands of reconstructing high‐resolution images, requiring substantial computational resources and time.

Beyond the computational bottlenecks encountered during image reconstruction, traditional SPI methods also face severe hardware‐level scalability issues during the data acquisition phase. In conventional HSI, as resolution increases, the required number of patterns grows quadratically, leading to a rapid increase in requisite memory and modulation time. For instance, the limited onboard memory of commercial DMDs, such as those controlled by the V‐7001 driver board, makes it challenging to modulate targets larger than 256×256 pixels. While 1D single‐pixel imaging (1D‐SPI) [29] achieves an N‐fold reduction in basis patterns, its methodology relies on mechanical translation to scan the target image row‐by‐row. This approach inherently inflates the total number of modulations; for every row the target moves, the spatial light modulator must re‐project the entire set of patterns. Consequently, imaging a 768×512 resolution target requires 1D‐SPI to modulate 1,024 patterns 512 times, culminating in a total modulation time that remains at 52.4 s even with a DMD operating at 10 kHz.

To fundamentally circumvent this row‐by‐row modulation penalty and the memory constraints of conventional HSI, our proposed HMS‐SPI employs an innovative opto‐electro‐mechanical co‐design that decouples the 1D spatial encoding from the physical longitudinal scanning process. During the projection of a single DMD basis pattern, a line‐shaped laser, facilitated by a synchronously triggered high‐speed galvanometer, completes one continuous periodic scan across the entire target image. Instead of capturing a single integrated intensity value per pattern, HMS‐SPI utilizes a high‐speed oscilloscope to acquire the continuous time‐series waveform of the reflected light. This paradigm shift enables the system to collect the spatial information of all columns within a single physical scanning cycle. Consequently, HMS‐SPI keeps the total number of modulations constant while reducing the number of projected patterns, resulting in an authentic N‐fold acceleration in both data throughput and modulation time. As indicated in Table 1, this method significantly enhances DMD utilization, maintaining a distinct advantage in imaging speed even with low‐frame‐rate models, and conserving computational resources.

**TABLE 1:** Comparison of HSI, 1D‐SPI, and HMS‐SPI parameters across different resolutions.

Resolution	128×128		256×256		512×512	
Parameter	Pattern	Modulation time	Pattern	Modulation time	Pattern	Modulation time
HSI	32768	3.28s (10k Hz)	131072	13.11s (10k Hz)	524288	52.43s (10k Hz)
		1.49s (22k Hz)		5.96s (22k Hz)		23.83s (22k Hz)
1D‐SPI	256	3.28s (10k Hz)	512	13.11s (10k Hz)	1024	52.43s (10k Hz)
		1.49s (22k Hz)		5.96s (22k Hz)		23.83s (22k Hz)
HMS‐SPI	256	3.20s (80 Hz)	512	6.40s (80 Hz)	1024	12.80s (80 Hz)
		1.00s (256 Hz)		2.00s (256 Hz)		4.00s (256 Hz)

### 2.3 Experiments

**FIGURE 4:**
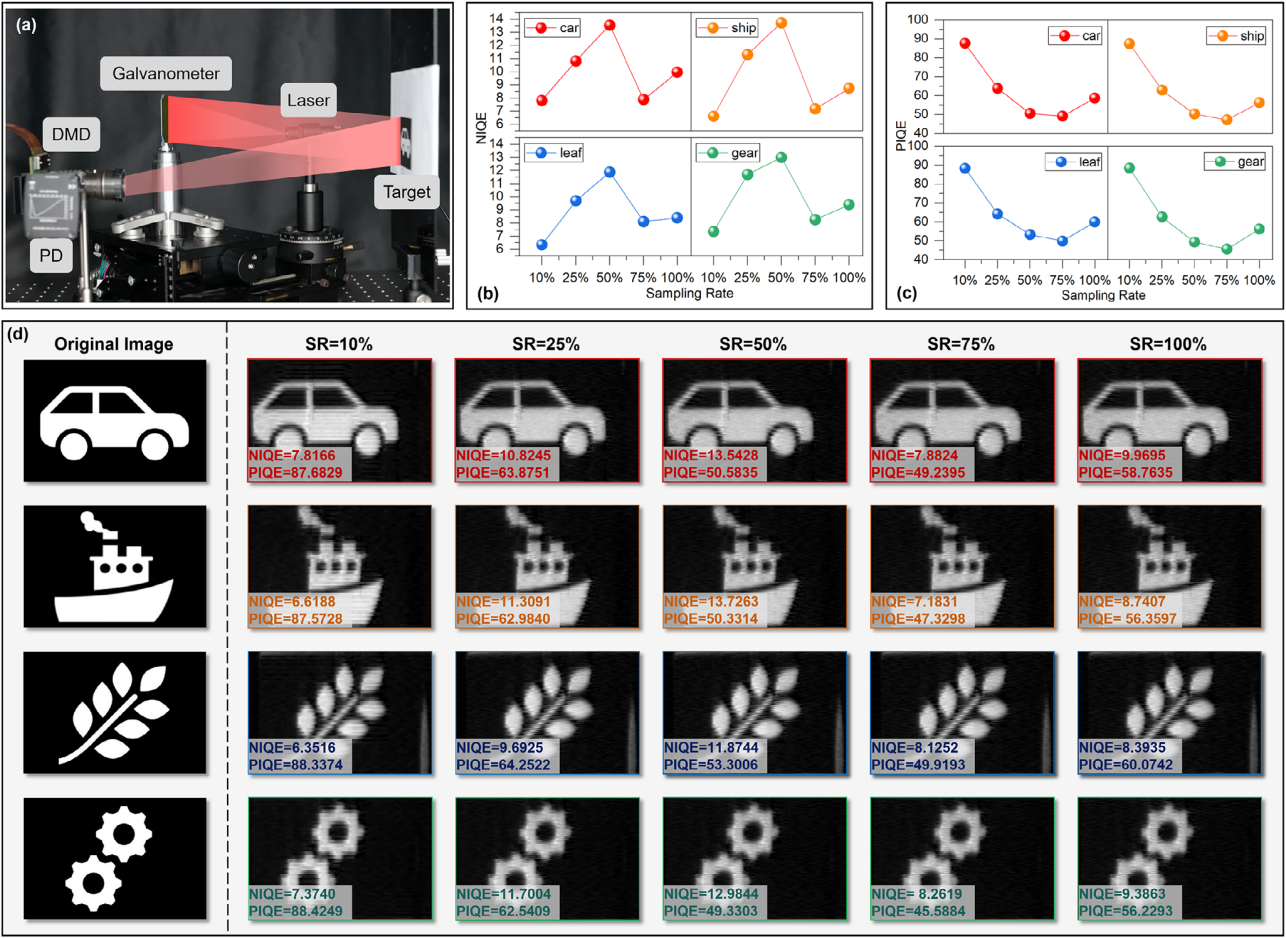
Hadamard matrix slicing single‐pixel imaging. (a) The experimental setup for laser illumination. (b) and (c) Comparison of the NIQE and PIQE results. (d) Image reconstruction results for different target images with a resolution of 768×512 pixels at various sampling rates.

In the experiment, images of a “car,” “ship,” “leaf,” and “gear,” each with a resolution of 768×512, are reconstructed at various sampling rates using a DMD operating at 80 Hz. The reconstructed images are presented in Figure 4d. At full sampling, each reconstruction requires only 1,024 patterns, and with the DMD's modulation frequency of 80 Hz, the total modulation time for an entire image is only 12.8 s. Furthermore, this method overcomes the conventional limitation of HSI, which is typically restricted to reconstructing images with dimensions that are integer powers of two, thereby enabling the reconstruction of rectangular target images.

The quality of the reconstructed images is assessed using the Natural Image Quality Evaluator (NIQE) and the Perception‐based Image Quality Evaluator (PIQE), with the results shown in Figure 4b,c. The quality assessment metrics are detailed in Note S5. Theoretically, a 100% sampling rate should provide the most complete image information, leading to the highest reconstruction quality. However, in physical experiments, the intrinsic coupling effect between the mechanical positional jitter of the galvanometer and high‐frequency Hadamard basis vectors is a critical factor. Because the spatial mapping of HMS‐SPI relies on the time‐axis scanning of the galvanometer, microsecond‐level velocity ripples during the mechanical sweep translate into sub‐pixel positional offsets.

To quantitatively evaluate the impact of this opto‐electro‐mechanical instability, we simulate the intrinsic jitter sensitivity of Walsh‐ordered Hadamard basis vectors (detailed in Note S6). The simulation reveals that low‐frequency basis vectors exhibit high robustness to spatial shifts, whereas high‐frequency basis vectors suffer from severe phase decoupling, causing the projection error to increase linearly with spatial frequency. As the sampling rate increases from 75% to 100%, the system is forced to employ the highest‐frequency basis vectors. The mechanical coupling noise and cumulative error introduced by these additional high‐frequency measurements exceed the highly sparse effective signal they provide. This degrades the overall image quality and induces subtle distortions and artifacts in the reconstructed image. Consequently, the 75% sampling rate establishes the optimal trade‐off between spatial resolution and mechanical noise tolerance, achieving the best reconstruction quality.

The experimental results demonstrate that the proposed method significantly reduces the number of required patterns, shortens the modulation time, and consequently enhances the imaging speed. In conventional HSI, reconstructing a 512×512 image requires 524,288 patterns; even with a DMD modulation frequency of 20 kHz, the total modulation time for the entire image is 26.2 s. Our method reduces the number of modulation patterns by a factor of N. Consequently, reconstructing a 512×512 image requires only 1,024 patterns, and theoretically, at a modulation frequency of 20 kHz, the potential image modulation time significantly decreases to 51.2 milliseconds.

## 3 Discussion

Inspired by linear array detectors, this study proposes HMS‐SPI, which establishes an efficient 1D scanning imaging paradigm. By integrating the advantages of optical multiplexing with periodic galvanometer scanning, this method successfully circumvents the inherent bottlenecks associated with 2D global modulation in traditional SPI. Although reducing the encoding dimensionality from $N^{2}$ to N mathematically alters the scale of spatial multiplexing, the orthogonal multiplexing advantage is strictly preserved along the 1D slice. Simultaneously, the concentrated optical power from the linear laser source maintains a high signal‐to‐noise ratio, effectively compensating for the reduced modulation area. This paradigm shift not only preserves the superior adaptability of single‐pixel detectors under low‐light and broad‐spectrum conditions but also endows them with high‐throughput data acquisition capabilities comparable to push‐broom linear array detectors. Consequently, it fundamentally resolves the technical challenge wherein sampling time scales quadratically with resolution, achieving an order‐of‐magnitude enhancement in imaging efficiency.

It is worth noting that the proposed linear‐array‐inspired scanning paradigm is not exclusively confined to HSI. The core philosophy of decoupling 2D spatial modulation into 1D lateral encoding, combined with physical longitudinal scanning, possesses broad generalizability. Consequently, this framework can be seamlessly extended to Fourier Single‐Pixel Imaging (FSPI). By substituting the 1D Hadamard slices with 1D sinusoidal basis vectors, Fourier slice imaging can be readily achieved. This adaptability further underscores the potential of the proposed paradigm as a general‐purpose accelerator applicable to various single‐pixel imaging modalities based on orthogonal bases.

In terms of implementation, HMS‐SPI demonstrates exceptional spatiotemporal utilization. Through the co‐design of a unique matrix slicing algorithm and galvanometer scanning, the system compresses the number of modulation patterns for a 768×512 resolution image to merely 1,024, while maintaining full sampling conditions. This extremely low data throughput significantly mitigates the on‐board memory consumption and pre‐loading latency of the DMD, overcoming the storage bandwidth limitations of commercial DMDs in high‐resolution imaging. Furthermore, this method liberates traditional HSI from strict constraints regarding square fields of view and integer‐power‐of‐two resolutions, enabling the flexible and direct reconstruction of rectangular regions with arbitrary aspect ratios. Such flexibility, combined with the potential to complete modulation within microsecond timescales, offers a robust solution for time‐critical scenarios such as industrial inspection and remote sensing.

Although HMS‐SPI achieves a qualitative leap in speed and efficiency, as an opto‐electro‐mechanical system, its imaging quality remains subject to the constraints of physical implementation. Experimental results indicate that speckle noise introduced by laser coherence and minor positioning errors from mechanical galvanometer scanning are the primary factors affecting the signal‐to‐noise ratio. The current system speed is capped at a frame rate of 80 Hz; however, this limitation stems primarily from the response constraints of the mechanical galvanometer employed, rather than the methodology itself. Theoretically, if high‐speed scanning mechanisms such as polygon mirrors or resonant galvanometers that align with the 20 kHz modulation frequency of the DMD are adopted, the modulation time of the entire imaging process is no longer constrained by mechanical motion. This implies that for high‐resolution images, the modulation duration is drastically compressed to the millisecond scale, which fully unleashes the high‐speed capabilities of the DMD and enables the realization of ultra‐high‐speed real‐time imaging.

## 4 Experimental Section

Experimental Setup: The photograph of the experimental setup is presented in Figure S2. The entire setup for the HMS‐SPI experiment utilized an industrial line‐generating laser source (Customized, Shenzhen Yuanda Laser Technology Co., Ltd.) in conjunction with a high‐speed galvanometer mirror (RC3808, Jiangsu Jinhaichuang Technology Co., Ltd.) to perform column‐by‐column scanning of the target. The laser source projects a linear beam onto the galvanometer, which was driven by a stable, continuous triangular wave signal to generate a constant and periodic optical scanning region. The influence of driving waveform characteristics on the scanning and sampling processes is elaborated in Note S4, where experimental results under alternative driving signals are also compared.

During each scan, the spatial information of the target was encoded into the laser beam, which was subsequently reflected onto a DMD (ViALUX V‐7001). To ensure precise modulation of the target information, the DMD was externally triggered by a 3 V square wave signal from a signal generator (Tektronix AFG2021), synchronized with the galvanometer. The reflected light intensity, modulated by encoding patterns preset on the DMD, was captured by a fixed detector (Thorlabs PDA100A2). This optical signal was subsequently converted into an electrical signal and synchronously acquired by an oscilloscope (Rohde & Schwarz RTO64). The synchronization design of the experimental apparatus is detailed in Note S3.

Computational Environment: All numerical simulations and image reconstructions presented in the main text were executed using MATLAB R2023b on a standard laptop computer equipped with a 6‐core, 12‐thread CPU (2.10 GHz base clock), and 16 GB of RAM. Additionally, the specific simulations detailed in Note S6 are performed using Python 3.9.

## Conflicts of Interest

The authors declare no conflicts to interest.

## Supporting information


**Supporting File**: advs75643‐sup‐0001‐SuppMat.pdf.

## Data Availability

Data supporting the findings of this study are available from the corresponding author upon reasonable request.

## References

[1] S. A. Taylor , “CCD and CMOS Imaging Array Technologies: Technology Review,” Xerox Research Centre Europe, Cambridge, UK, Tech. Rep. EPC‐1998‐106 (1998).

[2] S. Y. Fu , “Design of High‐Speed Data Acquisition System for Linear Array CCD Based on FPGA,” Procedia Computer Science 166 (2020): 414–418.

[3] S. Lv , T. Tang , J. Chen , X. Shi , and Y. Liu , “Enhancing Full‐Color Single‐Pixel Imaging: Integrating Variable Density Sampling with Hyper‐Laplacian Priors,” Applied Physics Letters 124, no. 13 (2024): 133301.

[4] G. M. Gibson , S. D. Johnson , and M. J. Padgett , “Single‐Pixel Imaging 12 Years On: A Review,” Optics Express 28, no. 19 (2020): 28190–28208. 32988095 10.1364/OE.403195

[5] M. P. Edgar , G. M. Gibson , and M. J. Padgett , “Principles and Prospects for Single‐Pixel Imaging,” Nature Photonics 13, no. 1 (2019): 13–20.

[6] C. Zhou , T. Tian , C. Gao , W. Gong , and L. Song , “Multi‐Resolution Progressive Computational Ghost Imaging,” Journal of Optics 21, no. 5 (2019): 055702.

[7] M. J. Sun and J. M. Zhang , “Single‐Pixel Imaging and Its Application in Three‐Dimensional Reconstruction: A Brief Review,” Sensors 19, no. 3 (2019): 732. 30754728 10.3390/s19030732 PMC6387278

[8] J. Cheng and S. Han , “Incoherent Coincidence Imaging and Its Applicability in X‐Ray Diffraction,” Physical Review Letters 92, no. 9 (2004): 093903. 15089466 10.1103/PhysRevLett.92.093903

[9] M. Chen , H. Wu , R. Wang , et al., “Computational Ghost Imaging with Uncertain Imaging Distance,” Optics Communications 445 (2019): 106–110.

[10] D. Zhan , H. Wang , J. Lin , et al., “Image Denoising and Deringing for Fourier Single‐Pixel Imaging Based on Upgraded Weighted Nuclear Norm Minimization,” Optics Communications 550 (2024): 130011.

[11] F. Ferri , D. Magatti , L. Lugiato , and A. Gatti , “Differential Ghost Imaging,” Physical Review Letters 104, no. 25 (2010): 253603. 20867377 10.1103/PhysRevLett.104.253603

[12] G. A. Howland , D. J. Lum , M. R. Ware , and J. C. Howell , “Photon Counting Compressive Depth Mapping,” Optics Express 21, no. 20 (2013): 23822. 24104293 10.1364/OE.21.023822

[13] B. Sun , M. P. Edgar , R. Bowman , et al., “3D Computational Imaging with Single‐Pixel Detectors,” Science 340, no. 6134 (2013): 844–847. 23687044 10.1126/science.1234454

[14] M. J. Sun , M. P. Edgar , G. M. Gibson , et al., “Single‐Pixel Three‐Dimensional Imaging with Time‐Based Depth Resolution,” Nature Communications 7, no. 1 (2016): 12010. 10.1038/ncomms12010 PMC5512623 27377197

[15] D. B. Phillips , M. J. Sun , J. M. Taylor , et al., “Adaptive Foveated Single‐Pixel Imaging with Dynamic Supersampling,” Science Advances 3, no. 4 (2017): e1601782. 28439538 10.1126/sciadv.1601782 PMC5400451

[16] Q. Guo , H. Chen , Y. Wang , et al., “High‐Speed Compressive Microscopy of Flowing Cells Using Sinusoidal Illumination Patterns,” IEEE Photonics Journal 9, no. 1 (2017): 1–11.

[17] B. Jalali and K. Goda , “Photonic Time‐Stretch: From World's Fastest Digitizer to the World's Fastest Camera,” in 2010 IEEE International Topical Meeting on Microwave Photonics (2010), 175–176.

[18] K. Goda , K. Tsia , and B. Jalali , “Serial Time‐Encoded Amplified Imaging for Real‐Time Observation of Fast Dynamic Phenomena,” Nature 458, no. 7242 (2009): 1145–1149. 19407796 10.1038/nature07980

[19] K. K. Tsia , K. Goda , D. Capewell , and B. Jalali , “Performance of Serial Time‐Encoded Amplified Microscope,” Optics Express 18, no. 10 (2010): 10016–10028. 20588855 10.1364/OE.18.010016

[20] K. Goda , A. Ayazi , D. R. Gossett , et al., “High‐Throughput Single‐Microparticle Imaging Flow Analyzer,” Proceedings of the National Academy of Sciences of the United States of America 109, no. 29 (2012): 11630–11635. 22753513 10.1073/pnas.1204718109 PMC3406874

[21] A. C. Chan , A. K. Lau , K. K. Wong , E. Y. Lam , and K. K. Tsia , “Arbitrary Two‐Dimensional Spectrally Encoded Pattern Generation–A New Strategy for High‐Speed Patterned Illumination Imaging,” Optica 2, no. 12 (2015): 1037–1044.

[22] C. Lei , B. Guo , Z. Cheng , and K. Goda , “Optical Time‐Stretch Imaging: Principles and Applications,” Applied Physics Reviews 3, no. 1 (2016): 011102.

[23] M. Shalaby , M. Peccianti , D. G. Cooke , C. P. Hauri , and R. Morandotti , “Temporal and Spectral Shaping of Broadband Terahertz Pulses in a Photoexcited Semiconductor,” Applied Physics Letters 106, no. 5 (2015): 051110.

[24] C. Zhou , X. Liu , Y. Feng , et al., “Real‐Time Physical Compression Computational Ghost Imaging Based on Array Spatial Light Field Modulation and Deep Learning,” Optics and Lasers in Engineering 156 (2022): 107101.

[25] Y. Qin , L. H. Li , Z. Yu , et al., “Ultra‐High‐Performance Amorphous ${\mathrm{Ga}}_{2}$ $O_{3}$ Photodetector Arrays for Solar‐Blind Imaging,” Advanced Science 8, no. 20 (2021): 2101106. 34390217 10.1002/advs.202101106 PMC8529488

[26] G. Martín , J. M. Bioucas‐Dias , and A. Plaza , “HYCA: A New Technique for Hyperspectral Compressive Sensing,” IEEE Transactions on Geoscience and Remote Sensing 53, no. 5 (2014): 2819–2831.

[27] A. Béché , B. Goris , B. Freitag , and J. Verbeeck , “Development of a Fast Electromagnetic Beam Blanker for Compressed Sensing in Scanning Transmission Electron Microscopy,” Applied Physics Letters 108, no. 9 (2016): 093103.

[28] H. Zhang , J. Cao , C. Zhou , H. Yao , and Q. Hao , “Explicit Compression Degradation Estimations for Low‐Sampling Single‐Pixel Imaging Using Hadamard Basis,” Advanced Science 13, no. 9 (2026): e12655. 41319281 10.1002/advs.202512655 PMC12904066

[29] M. Ma , Y. Gao , J. Hou , et al., “One‐Dimensional Modulation Single‐Pixel Imaging: Exceeding Spatial Light Modulator Resolution,” Optics and Lasers in Engineering 176 (2024): 108071.

[30] T. B. Pittman , Y. H. Shih , D. V. Strekalov , and A. V. Sergienko , “Optical Imaging by Means of Two‐Photon Quantum Entanglement,” Physical Review A 52, no. 5 (1995): R3429–R3432. 10.1103/physreva.52.r3429 9912767

[31] J. H. Shapiro , “Computational Ghost Imaging,” Physical Review A 78, no. 6 (2008): 061802.

[32] O. Katz , Y. Bromberg , and Y. Silberberg , “Compressive Ghost Imaging,” Applied Physics Letters 95, no. 13 (2009): 131110.

[33] L. Yin , H. Zhan , W. Tang , et al., “Ghost Imaging under Direct Sunlight Conditions Using FADOF,” Applied Physics Letters 124, no. 8 (2024): 084002.

[34] M. Lyu , W. Wang , H. Wang , et al., “Deep‐Learning‐Based Ghost Imaging,” Scientific Reports 7, no. 1 (2017): 17865. 29259269 10.1038/s41598-017-18171-7 PMC5736587

[35] T. Shimobaba , Y. Endo , T. Nishitsuji , et al., “Computational Ghost Imaging Using Deep Learning,” Optics Communications 413 (2018): 147–151.

[36] C. F. Higham , R. Murray‐Smith , M. J. Padgett , and M. P. Edgar , “Deep Learning for Real‐Time Single‐Pixel Video,” Scientific Reports 8, no. 1 (2018): 2369. 29403059 10.1038/s41598-018-20521-y PMC5799195

[37] K. Komuro , T. Nomura , and G. Barbastathis , “Deep Ghost Phase Imaging,” Applied Optics 59, no. 11 (2020): 3376–3382. 32400448 10.1364/AO.390256

[38] L. T. Meng , P. Jia , H. H. Shen , et al., “Sinusoidal Single‐Pixel Imaging Based on Fourier Positive‐Negative Intensity Correlation,” Sensors 20, no. 6 (2020): 1674. 32192203 10.3390/s20061674 PMC7146428

[39] J. Tan , T. Zou , H. Chen , W. Su , and Z. He , “Spectral Division Multiplexing‐Based Region Projection for 3D Measurement under Mutual Reflection,” Optics Letters 51, no. 2 (2026): 488–491. 41538873 10.1364/OL.574895

[40] W. Su , J. Tan , Z. He , Z. Lin , and H. Liang , “Separable Hadamard Single‐Pixel Imaging for High‐Resolution Reconstruction,” Optics Letters 50, no. 24 (2025): 7468–7471. 41396906 10.1364/OL.576371

[41] M. Bevilacqua , A. Roumy , C. Guillemot , and M. Line Alberi Morel , “Low‐Complexity Single‐Image Super‐Resolution Based on Nonnegative Neighbor Embedding,” in Proceedings of the British Machine Vision Conference (2012), 135.1–135.10.

[42] W. K. Yu , “Super Sub‐Nyquist Single‐Pixel Imaging by Means of Cake‐Cutting Hadamard Basis Sort,” Sensors 19, no. 19 (2019): 4122. 31548513 10.3390/s19194122 PMC6806058

