# Supplementary material for: Hadamard Matrix Slicing Single‐Pixel Imaging: Establishing a Linear‐Array‐Inspired Paradigm for N‐fold Acceleration Single‐Pixel Imaging
Source: Adv Sci (Weinh). 2026 May 12;13(43):e75643. doi: 10.1002/advs.75643 (PMC13335940; doi:10.1002/advs.75643)
Supplement: Supplementary file 1 — Supporting File: advs75643‐sup‐0001‐SuppMat.pdf. [file ADVS-13-e75643-s001.pdf]

Supplementary Material for

Hadamard Matrix Slicing Single-Pixel Imaging: Establishing a Linear-Array-Inspired Paradigm for *N*-fold Acceleration Single-Pixel Imaging

Xiaoxue Li,[1] Jieting Hou,[1] Qizheng Zhao,[1] Yiqi Jia,[2] Xiang Zhong,[1] Mengchao Ma,[1,*] Huaxia Deng,[2,*] Xinglong Gong,[2] and Ziwei Wang,[3,*]

[1]Anhui Province Key Laboratory of Measuring Theory and Precision Instrument, School of Instrument Science and Opto-Electronics Engineering, Hefei University of Technology, Hefei, Anhui, 230009, People's Republic of China.

[2]CAS Key Laboratory of Mechanical Behavior and Design of Materials, Department of Modern Mechanics, University of Science and Technology of China, Hefei, Anhui, 230027, People's Republic of China.

[3]School of Engineering, Lancaster University, Lancaster LA1 4YW, United Kingdom.

*Corresponding author. Email: mmchao@hfut.edu.cn, hxdeng@ustc.edu.cn, z.wang82@lancaster.ac.uk

This document provides supplementary material to "Hadamard Matrix Slicing Single-Pixel Imaging: Establishing a Linear-Array-Inspired Paradigm for *N*-fold Acceleration Single-Pixel Imaging".

This PDF file includes:

Supplementary Note 1 to Note 6

Figures S1 to S6

Table S1

Supplementary Note 1: Specific reconstruction times for different image resolutions

In the conventional single-pixel imaging (SPI) process, each projected frame from the spatial light modulator represents a single modulation of the target scene. The complete set of measurements is acquired by sequentially projecting numerous frames, a procedure that necessitates an intensive modulation process from the digital micromirror device (DMD). Consequently, the large number of required mask patterns leads to a prolonged acquisition time. Although conventional Hadamard single-pixel imaging (HSI) employs an undersampling strategy to reduce the number of measurements, this is often at the expense of reconstruction quality. Moreover, as the image resolution increases, both the imaging time and computational demands escalate sharply. To address this, we conduct MATLAB numerical simulations to compare the computational times of HSI and our proposed Hadamard Matrix Slicing Single-Pixel Imaging (HMS-SPI). The specific reconstruction times for various resolutions and the performance of HMS-SPI in reconstructing a rectangular target area are detailed in Table S1.

Table S1. Reconstruction times for images at various resolutions.

Resolution	32×32	64×64	128×128	256×256	512×512
HSI(s)	0.003643	0.005219	3.881047	36.956198	840.097071
HMS-SPI(s)	0.009083	0.033002	0.148074	0.835149	5.360375
Resolution	128×500	256×500	512×500	1024×500	2048×1000
HMS-SPI(s)	0.377856	1.215265	0.148074	19.388048	168.962745

It is observed that the computational time for HSI increases dramatically starting from a resolution of 64×64. The comparative results demonstrate that HMS-SPI holds a significant advantage in terms of reconstruction speed.

Supplementary Note 2: Optical pathway simulation and design

The optical path simulation is performed using Zemax to ensure the feasibility of the proposed scheme. The optical paths are preset differently depending on the size of the reconstructed target as well as the requirements for the galvanometer position and oscillation amplitude. A simulation of the Zemax optical path for one case is shown in Figure S1.

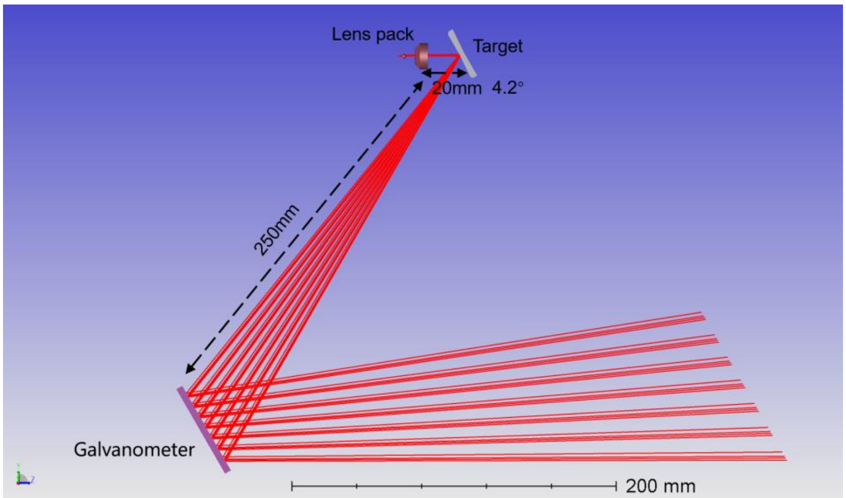

Figure S1: Scanning HMS-SPI optical path.

The uniform line laser integrated by the lens group (plano-convex lens and Powell prism) has a divergence angle of 30° in the vertical direction. As shown in Figure S1, the projector device consists of a lens group and a digital micromirror array located at the phase surface at the end of the optical path. The experiment is carried out in the forward direction of the optical path after first calibrating the target position in the reverse direction of the single-pixel optical path. The laser beam is directed onto the center axis of the galvanometer to form an orthogonal geometric system in space. To accurately and completely scan the target, the galvanometer needs to be rotated by 4.2°, and only beam changes per 0.7° are shown in the simulation. Due to the structural characteristics of the system and the requirement for time synchronization, it is only necessary to ensure that the scanning beam is within the starting interval in the scanning direction.

Supplementary Note 3: Synchronized design of experimental equipment

In the experiment, the driving of the galvanometer, the external triggering of the DMD, and the acquisition of the synchronization pulse (Sync Pulse) and the photodetector output voltage are fully synchronized. The external trigger signal required for the DMD and the triangular wave signal driving the galvanometer are synchronized by a signal generator (Tektronix AFG2021). A square wave signal serves as the external trigger, where its rising edge triggers the DMD to initiate pattern projection. The frequency and voltage magnitude of the triangular wave correspond to the galvanometer's rotational speed and deflection angle. As shown in the experimental setup in Figure S2, the photodetector (PD) is mounted onto the DMD via an adapter, a configuration that optimizes light intensity collection and reduces stray laser interference.

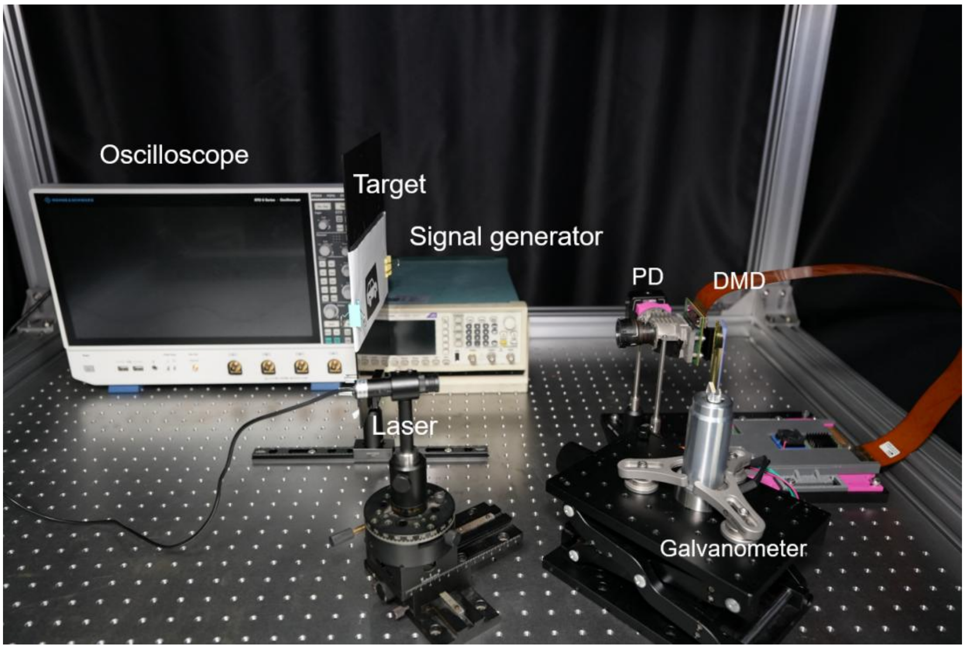

Figure S2: Photograph of the experimental setup.

The accuracy of the modulation process in a high-speed single-pixel imaging system is critically dependent on the synchronization between the frame rate of the DMD and the speed of the galvanometer. To achieve this, high-precision driving signals are employed. The control waveform parameters for both the DMD and the galvanometer are calculated based on the system's target frame rate to ensure synchronization. For a target resolution of $N \times M$ pixels, the measurement of M horizontal pixels is completed within a single deflection period of the galvanometer. Consequently, the period of the triangular wave signal driving the galvanometer must be identical to that of the square wave trigger for the DMD. The required voltage of this triangular wave is calculated based on the optical path length between the galvanometer and the target. Once configured, the two drive signal outputs from the signal generator are connected to the respective control inputs of the hardware devices to enable synchronous triggering.

The DMD is configured such that for trigger periods exceeding 10 ms, it outputs a Sync Pulse signal concurrently with the display of each extended one-dimensional encoding pattern. This Sync Pulse is transmitted to the acquisition device (oscilloscope). The display duration of each mask pattern (the "bright frame") is set to match the high-state duration of this Sync Pulse. The oscilloscope is configured to trigger autonomously upon detecting this signal, thereby initiating the acquisition of the modulated image signal. A schematic diagram of the hardware connections is presented in Figure S3.

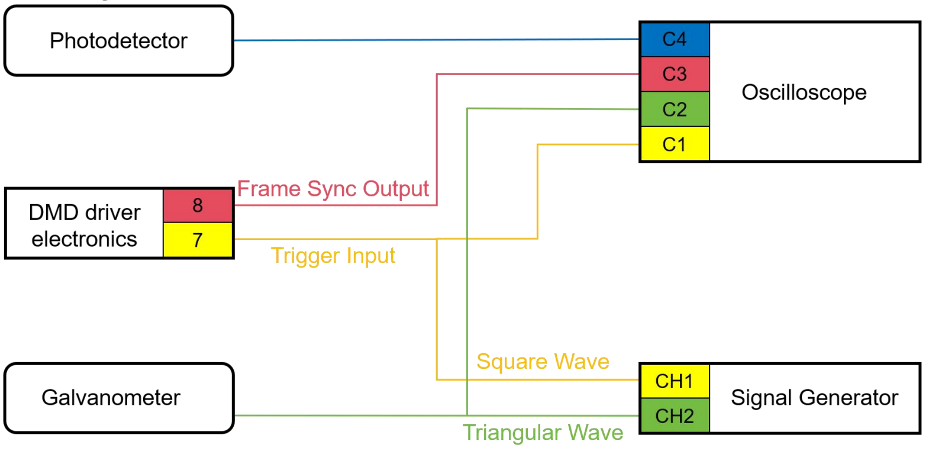

Figure S3: Hardware device connection schematic.

As shown in Figure S4, (a) displays the waveform signals of the four oscilloscope channels within a complete image acquisition cycle, and (b) provides the corresponding waveforms with an adjusted vertical display scale. Text labels and arrows of corresponding colors mark the waveform signals of the four channels, including the synchronization output pulse signal, the DMD drive signal, the galvanometer drive signal, and the image acquisition signal. Synchronization between devices relies on the one-to-one correspondence of signal periods to achieve precise scanning control.

(a)

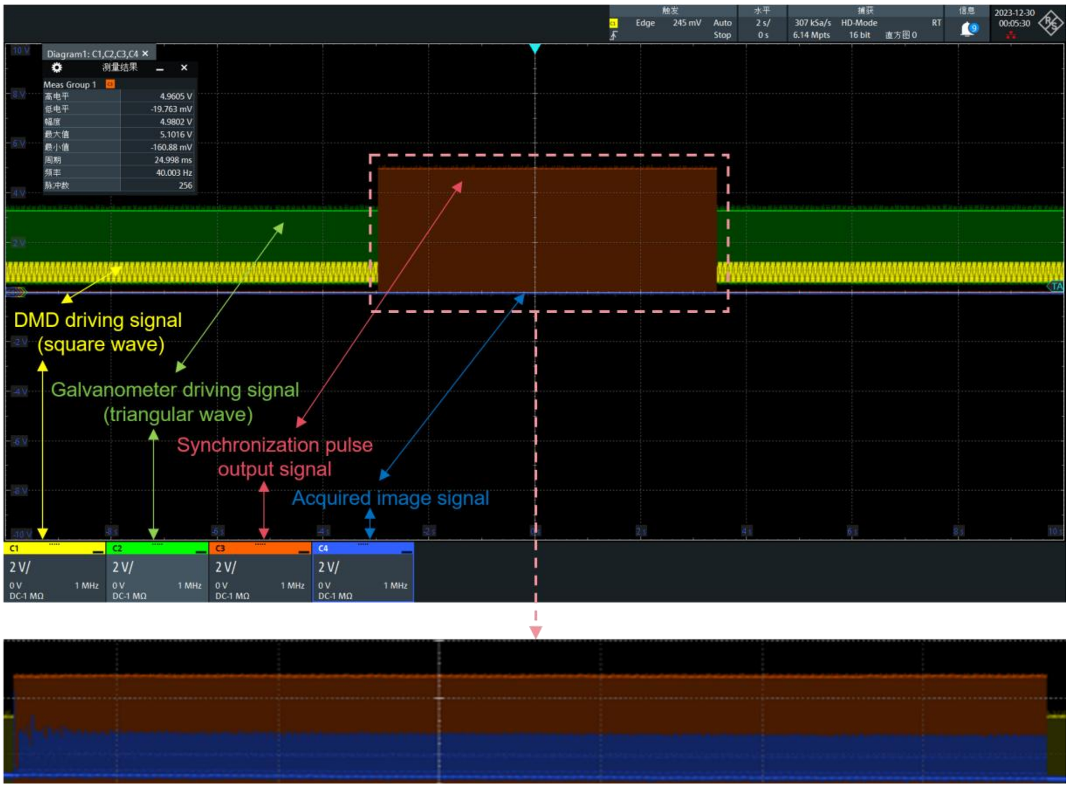

(b)

Figure S4: Oscilloscope waveform signals. (a) Waveform signals of the four oscilloscope channels within a complete image acquisition cycle. (b) Image acquisition waveform signals with an adjusted vertical display scale.

Supplementary Note 4: Comparison of reconstructed images under different galvanometer driving signals

The derivative of the driving waveform represents the variation in the scan rate, while its integration in the time domain corresponds to the sampling process. The effects of image reconstruction under the other two driving signal strategies, excluding the triangular waveform signal, are experimentally verified. Due to a minor optical path difference during the scanning process, different waveforms yield distinct results. First, a full sine wave with a non-uniform rate variation is selected to compensate for the optical path difference. However, the faster scanning rate in the middle region fails to completely eliminate this difference because the scanning velocity at the peaks and valleys drops to zero. Therefore, the reconstructed image exhibits an obvious squeezing artifact, as shown in the yellow dashed box in Figure S5(d). Alternatively, a partial sine wave is employed to image only a specific region, aiming to reduce pixel stacking. In this case, however, trailing artifacts appear at the image edges, as shown inside the yellow dashed box in Figure S5(c). Ultimately, multiple experiments demonstrate that the triangular wave driving signal provides a constant slope and minimizes the scanning angle over the focal length range, making it optimally suited for the proposed scanning model. Consequently, the triangular wave signal is exclusively utilized to drive the galvanometer for scanning in all experiments presented in the main text. Figure S5 summarizes the reconstruction results of a 256×330 target ("gear") under different galvanometer driving signals and sampling rates, with a DMD frame rate of 40 Hz.

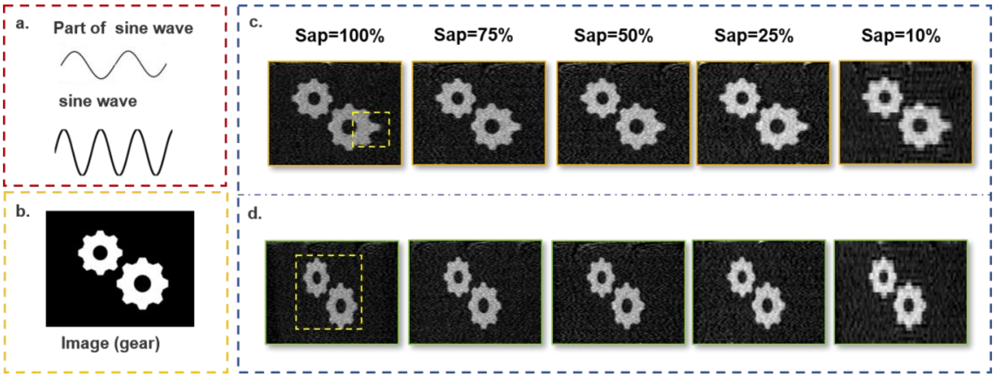

Figure S5: Comparison of reconstructed images under different galvanometer driving signals. (a) Galvanometer driving waveforms, including a partial sine wave and a full sine wave. (b) The original target image ("gear"). (c) Image reconstruction results using a partial sine wave, where trailing artifacts appear at the image edges due to attempts to reduce pixel stacking. (d) Image reconstruction results using a full sine wave driving signal, showing an obvious squeezing artifact because the non-uniform scanning rate fails to completely eliminate the optical path difference.

Supplementary Note 5: Detailed explanation of quality assessment indicators

In this work, the quality of simulated reconstruction results is evaluated using the Structural Similarity Index (SSIM) and Peak Signal-to-Noise Ratio (PSNR), while for experimental results, the assessment is conducted using the Natural Image Quality Evaluator (NIQE) and Perception based Image Quality Evaluator (PIQE). A detailed description of these four quality assessment metrics is provided below:

Peak Signal-to-Noise Ratio (PSNR)

PSNR is a full-reference image quality assessment (IQA) metric that quantifies reconstruction fidelity by measuring the ratio between the maximum possible pixel intensity and the power of the error signal. The error is defined by the pixel-wise differences between a reference image and its distorted counterpart, typically quantified by the Mean Squared Error (MSE). The core principle of PSNR is that it evaluates image quality based on the numerical deviation of pixel values. Specifically, it computes the MSE across the entire image and then expresses this error on a logarithmic decibel scale relative to the maximum signal power. A higher PSNR value indicates lower error and, therefore, a closer reconstruction to the reference image.

Structural Similarity Index Measure (SSIM)

SSIM is a full-reference IQA metric that evaluates distortions from a perceptual standpoint, focusing on the degradation of structural information. It models image degradation as a combination of three distinct factors: luminance, contrast, and structural loss, which are derived from local image statistics such as mean and variance. The core principle of SSIM is that the human visual system is highly sensitive to changes in structural content. Specifically, it compares local windows from the reference and distorted images to compute luminance, contrast, and structural similarity terms, which are then combined to form a local SSIM score. A final SSIM value is obtained by averaging these scores across the image, with a value closer to 1 indicating better preservation of structural content.

Natural Image Quality Evaluator (NIQE)

NIQE is a no-reference IQA metric that evaluates quality by comparing the statistical properties of a distorted image to those of a pre-established model of natural images. It relies on a set of features known as Natural Scene Statistics (NSS), which capture the characteristic regularities found in high-quality, undistorted images. The core principle of NIQE is that distortions disrupt these natural statistical regularities in a measurable way. Specifically, it extracts statistical features from the input image, fits them to a multivariate Gaussian model, and then calculates the distance between this model and a pre-trained model built from a high-quality image database. A lower NIQE score signifies that the image's statistics are closer to those of a natural scene, indicating better perceptual quality.

Perception based Image Quality Evaluator (PIQE)

PIQE is a no-reference IQA metric that quantifies distortion based on principles of human visual perception without requiring a reference image. It primarily identifies perceptually relevant artifacts, such as blocking artifacts and Gaussian noise, by analyzing the image's local characteristics. The core principle of PIQE lies in segmenting the image into high-activity and low-activity regions and assessing distortions that are most noticeable to the human eye. Specifically, it computes block-wise distortion scores and then intelligently pools them, assigning greater weight to perceptually significant regions, to produce a final quality score. A lower PIQE value indicates better perceptual quality with fewer noticeable artifacts.

Supplementary Note 6: Intrinsic jitter sensitivity of Hadamard basis vectors under mechanical scanning

In the proposed HMS-SPI system, target sampling employs a mechanical galvanometer that drives a line-shaped laser to perform continuous periodic scanning across the target object. Despite the high precision of the system, the dynamic scanning process inevitably introduces sub-pixel velocity nonlinearities and positional micro-jitters. Because single-pixel imaging relies on the integration of modulated light intensity, any spatial misalignment between the dynamic optical beam and the static DMD patterns directly translates into projection errors.

To quantitatively evaluate the impact of this optomechanical instability, we simulate the intrinsic jitter sensitivity of the Walsh-ordered Hadamard basis vectors. In this simulation, we introduce a sub-pixel positional jitter to characterize the mechanical mapping errors. The expected projection error is calculated as the absolute integration difference between the ideal basis vector and its spatially shifted counterpart.

Setting the sub-pixel positional jitter to 0.2 pixels, the simulation results presented in Figure S6 demonstrate that the expected projection error exhibits a positive linear correlation with the spatial frequency of the Hadamard patterns. Specifically, low-frequency bases exhibit high robustness, whereas high-frequency bases are highly sensitive to mechanical fluctuations. Low-frequency patterns, primarily corresponding to the 0%–75% sampling interval, feature fewer spatial transitions between +1 and -1. This characteristic renders them highly robust against sub-pixel spatial shifts, where slight misalignments cause only minor integration errors at a limited number of boundaries. Conversely, high-frequency patterns utilized in the 75%–100% sampling range alternate rapidly and possess dense transition edges. Consequently, even minimal positional jitters generated by the galvanometer trigger extensive edge mismatches across the entire scanning line, rendering these high-sequency patterns exceptionally sensitive to mechanical fluctuations.

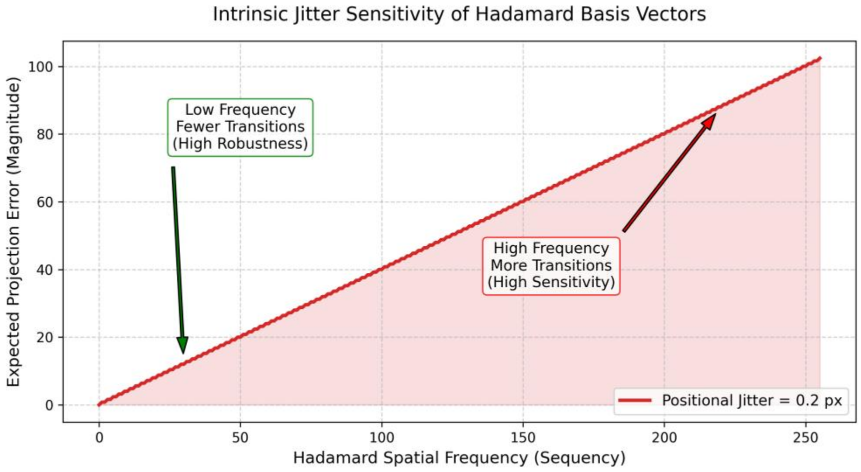

Figure S6: Simulation of the intrinsic jitter sensitivity of Walsh-ordered Hadamard basis vectors. The

plot demonstrates the linear relationship between the spatial frequency and the magnitude of the expected projection error under a sub-pixel positional jitter of 0.2 pixels.

This theoretical analysis perfectly elucidates the phenomena observed in the image reconstruction experiments under various sampling rates. When the sampling rate exceeds 75%, the mechanical mapping noise introduced by these ultra-high-frequency measurements outweighs the effective high-frequency spatial details they provide. The noise induced by sub-pixel optomechanical misalignments severely overwhelms the minute image signals that these high-frequency patterns are intended to capture. Therefore, within the proposed HMS-SPI paradigm, the 75% sampling threshold essentially dictates the optimal trade-off point between spatial resolution enhancement and mechanical jitter tolerance.
